# Oxidative stress markers in patient-derived non-cancerous cervical tissues and cells

**DOI:** 10.1038/s41598-020-76159-2

**Published:** 2020-11-04

**Authors:** Meghri Katerji, Maria Filippova, Yan Chen Wongworawat, Sam Siddighi, Sveta Bashkirova, Penelope J. Duerksen-Hughes

**Affiliations:** 1 Department of Basic Science, Loma Linda University School of Medicine, Loma Linda, CA 92354 USA; 2 Department of Obstetrics and Gynecology, Loma Linda University School of Medicine, Loma Linda, CA 92354 USA

**Keywords:** Cancer, Oncology

## Abstract

High-risk human papillomaviruses (HPV) are the causative agents of cervical cancer. However, not all infected women develop cervical cancer. Cervical tumorigenesis is characterized by a multifactorial etiology, with oxidative stress (OS) likely playing a major role. In addition to exogenous sources, metabolic processes also contribute to OS. In principle, variability in levels of cervical OS has the potential to influence the likelihood of conversion to cervical cancer. To ask whether such variability indeed existed, we assessed the levels of ROS and the oxidative DNA damage biomarker 8-oxodG in normal non-cancerous cervical tissues and cells obtained from women with uterovaginal pelvic organ prolapse following vaginal hysterectomy. We demonstrated five and ten-fold variability between tissues isolated from the transformation zone (TZ) and ectocervix (EC) of different women, respectively. Despite the greater variability (likely due to differences in tissue composition), the overall pattern of ROS levels in EC tissues mirrored those obtained in their corresponding TZ tissues. Our results also show that the levels of ROS in TZ tissues were always higher than or equal to those found in the respective EC tissues, providing a possible explanation for TZ tissue being the primary target for HPV infection and cervical carcinogenesis. Interestingly, primary keratinocytes isolated and cultured from these cervical specimens also displayed high variability in ROS levels, with some strongly mirroring the levels of ROS observed in their corresponding tissues, while others were less closely associated. Finally, we demonstrated that the levels of DNA damage mirrored the levels of ROS in the cultured primary cells. Understanding the factors and mechanisms that dispose certain individuals to develop cervical cancer has the potential to enable the development of approaches that make the conversion of HPV infection to cancer development even more rare.

## Introduction

Cervical cancer is the fourth most common cancer as well as the fourth leading cause of cancer-related death in women worldwide. Each year, it strikes nearly half a million women worldwide and claims a quarter of a million lives^1,2^. High risk human papillomavirus (HPV) infection is well-established as the causative agent of cervical cancer^3,4^. At least 85% of premalignant and 90% of malignant squamous lesions in the uterine cervix test positive for HPV DNA^5^. Infections with human papillomaviruses are extremely common; approximately 80 million Americans are currently infected, with another 14 million new infections occurring each year. In more than 90% of these cases, the infection is cleared by the immune system within two years, especially in younger women and adolescents^6–8^. However, a relatively small subset of infections persists, and of these, some progress to malignancy. In particular, approximately 0.3–0.5% of pap smear specimens are typically diagnosed as indicating carcinoma in situ^9^. This tells us that not all infected women develop cervical cancer, and that, in fact, the vast majority will not. Understanding the factors and mechanisms through which some, though not most, individuals develop cervical cancer has the potential to enable the development of approaches that make the conversion of HPV infection to cancer development even more rare.

Because HPV infection alone is not sufficient to induce cervical cancer, cervical tumorigenesis is clearly characterized by a multifactorial etiology^10^, with oxidative stress (OS) likely playing a major role in the process^11,12^. A number of clinical conditions have implicated OS as a contributory factor, including chronic inflammation, diabetes, atherosclerosis, ischemia–reperfusion injury, and of particular interest, malignancies of different origins^13–15^. In the case of cervical cancer, known risk factors in addition to HPV infection include smoking^16^, tar-exposure^17^, co-infection with other viruses such as herpes simplex virus-2^17^, co-infection with other STD, lifestyle, and diet^18^. Each of these factors can induce OS by one mechanism or another. For example, smoking was shown to induce OS by increasing the level of free radicals^19,20^. Infections and co-infections that induce inflammation also result in increases in the levels of reactive oxygen species (ROS), as the innate immune defense system utilizes the induction of OS as a powerful weapon against pathogens (reviewed in^21,22^). Elevated levels of reactive oxygen species (ROS) induce damage to DNA, lipids and proteins, inactivation of tumor suppressor genes, and enhanced expression of proto-oncogenes^14,23^. With regards to DNA, the ability of reactive nitrate and oxygen species to damage DNA, thereby leading to single and double-strand breaks, larger-scale damage and cancer, is well known^15^. For example, lungs of cigarette smokers contain two to three fold higher levels of the modified deoxynucleotide, 8-hydroxy-2′-deoxyguanosine (8-oxodG)^24^, which was shown to be induced by oxygen free radicals, resulting in inflammatory responses, fibrosis and tumor development^25^.

In addition to exogenous sources of OS such as smoking and infection, metabolic processes can also contribute to the level of OS in living cells. Under normal circumstances, homeostasis of ROS is maintained by several mechanisms, including the genetic and epigenetic regulation of genes coding for proteins that function in pro-and anti-oxidant systems^26,27^. Individual variability in the expression and/or function of such proteins and their regulators has the potential to translate into variability in levels of ROS, and in fact, the human population has been shown to be heterogeneous with regards to ROS levels^28–30^. While the role of exogenous factors in influencing the risk of cervical cancer is well documented^17,18^, available information on the influence of genetic/epigenetic factors on cervical cancer risk is fragmented. Most published studies have focused on finding correlations between particular genetic markers or mutations with cancer incidence by studying tissue that is already cancerous^31^. Interestingly, even the question of whether cervical cancer is or is not influenced by heredity is still in debate. Some consider that cervical cancer is not hereditary because the causative agent for cancer is HPV, which is the same for virtually all cases^32^. Another group believes that because genetic susceptibility to HPV exposure and/or infection appears to be important in determining the individual risk for developing HPV-mediated cancer, this cancer could be considered to have a hereditary component^33^.

As noted earlier, infection of cervical cells with high-risk HPV can lead to a significant health issue. Understanding the factors that play a major role in the process of HPV-mediated cervical carcinogenesis is therefore a clinically important question to address, since it enables the development of therapeutic strategies to prevent and/or treat carcinogenesis in HPV-infected individuals. In this study, we suggest that a high level of background ROS in cervical tissues, which may result from a combination of external influences as well as internal genetic/epigenetic factors, has the potential to contribute to the likelihood that a particular woman will develop cervical cancer. Importantly, accessibility of normal non-cancerous cervical samples from women with uterovaginal organ prolapse via vaginal hysterectomy gives us the advantage to assess this possibility. Hence, we characterized normal, non-cancerous cervical tissues for levels of ROS, and demonstrated five and ten-fold variability between tissues isolated from the transformation zone (TZ) and ectocervix (EC) of multiple women, respectively. In addition, primary keratinocytes were isolated and cultured from these tissue specimens, and also displayed high variability in ROS levels, with some strongly mirroring the levels of ROS observed in their corresponding tissues, while others were less closely associated. Finally, we demonstrated that the level of DNA damage mirrored the level of ROS in these patient-derived cultured cells.

## Materials and methods

### Collection of cervical specimens

Normal cervical specimens were collected from patients with uterovaginal pelvic organ prolapse following vaginal hysterectomy at the Loma Linda University Surgical Hospital. None of the women were diagnosed with HPV-related issues prior to surgery. Samples were anonymized to prevent linkage to identifiable individual data. Waste tissues not needed for clinical analysis were collected and placed into 0.9% normal saline solution and transported to the laboratory. Upon sample collection, each specimen was dissected in cold PBS under sterile cell culture conditions. The transformation zone (TZ) and ectocervical (EC) regions, labeled with black and white stitches by the surgeon, were separated based on the gross anatomy of the cervix (Supplementary Fig. 1), and divided into several parts for further use. The precise harvesting of TZ and EC samples from the surgical specimens were validated using Hematoxylin and Eosin histological staining. All methods were carried out in accordance with relevant guidelines and regulations.

### Measurement of ROS levels in cervical tissues

Specimens isolated from the TZ and EC were weighed and transferred to glass vials containing PBS for homogenization. The volume of PBS used for each sample was calculated to yield 200 mg of tissue in 1 ml of PBS. Homogenates were centrifuged at 10,000 rpm for 5 min at 4 °C, after which the supernatant was transferred to a new eppendorf tube, then snap-frozen in liquid nitrogen and stored at − 80 °C until use. The levels of reactive oxygen and nitrogen species (generally referred to as “ROS”) in these tissue homogenates were measured using the OxiSelect In Vitro ROS/RNS assay kit (Cell BioLabs, San Diego, CA) according to the manufacturer’s protocol. The assay employs a proprietary quenched fluorogenic probe, dichlorodihydrofluorescin DiOxyQ which becomes rapidly oxidized to the highly fluorescent 2′, 7′-dichlorodihydrofluorescein by hydrogen peroxide, peroxyl radical, nitric oxide and peroxynitrite anion. Therefore, the fluorescence intensity measured at 480 nm excitation/530 nm emission using a fluorometric plate reader is proportional to the total levels of ROS/RNS within the samples. Protein concentrations were measured with the Coomassie Plus Assay Reagent (Thermo Fisher Scientific, Rockford, IL) and were used for normalization. ROS/RNS levels are expressed as Relative Fluorescence Unit (RFU) per μg of protein.

### Isolation and culture of normal primary cervical keratinocytes

Primary keratinocytes from TZ regions were isolated using the protocol “Isolation, Primary Culture, and Cryopreservation of Human Keratinocytes” from Thermo Fisher Scientific (Life Technologies). Briefly, tissue sections were subjected to trypsin and dispase digestion, after which cells were retrieved and plated onto matrix-coated flasks in the presence of EpiLife media (LifeTechnology). After 2 days, feeder cells (NIH 3T3 mouse fibroblasts treated with 4 μg/ml of Mitomycin C (Sigma-Aldrich, St. Louis, MO) for 4 h) were added, and the media was replaced with keratinocyte growth media, E-media (DMEM supplemented with 22% Hams F12, 10% FBS, 1% Penicillin/Streptomycin, 0.2% Primocin, 0.5% Amphotericin B, 1.4 ng/ml Tri-iodo-thyronine, 40 ng/ml Hydrocortisone, 8.4 ng/ml Cholera toxin, 5 μg/ml Transferrin, 5 μg/ml Insulin, and 0.4 ng/ml Epidermal growth factor). To prevent keratinocyte differentiation, 10 μM of the ROCK pathway inhibitor, Y-27632 (Tocris, Bristol, UK), was included in the culture medium, allowing indefinite culturing of these cells.

### ROS level measurement in cultured keratinocytes

To limit technical variability between samples, all keratinocyte lines were passaged only a few times after isolation, enough to grow them to the appropriate cell density needed for analysis, and then were used for measuring ROS levels. Briefly, confluent flasks were washed with PBS, and fibroblast feeder cells were removed following trypsinization by diluted Trypsin (1:5). The attached keratinocytes were then trypsinized, collected and counted. 2.5 × 10^6^ cells were centrifuged at 2000 rpm for 5 min and washed with 1 mL PBS. The pellet was resuspended in 500 µl PBS and homogenized for 2 min. The sample was then centrifuged at 10,000 rpm at 4 °C for 5 min, and the supernatant was aliquoted in an eppendorf tube and stored at − 80 °C until use. ROS levels in these cell homogenates were then measured using the OxiSelect In Vitro ROS/RNS assay kit (Cell BioLabs, San Diego, CA) as previously described.

### DNA damage analysis in cultured keratinocytes

DNA damage was determined via the direct binding of fluorescein isothiocyanate (FITC)-labeled avidin to 8-oxodG residues in the genomic DNA. Briefly, primary cells were collected, washed twice with PBS, and fixed with 4% paraformaldehyde for 15 min. Cells were then washed three times with PBS, permeabilized with 75% ethanol and stored at − 20 °C until ready for experimental use.

All samples were washed, blocked, and incubated with 2 µg/ml Avidin-FITC (Thermo Fisher Scientific Life Technologies) for 1 h in the dark. After two washes, they were resuspended in PBS and analyzed by flow cytometry for fluorescence (excitation 495 nm, emission 515 nm) on a MACSQuant Analyzer 10 flow cytometer (Miltenyi Biotec Inc). A total of 10,000 events were measured per sample and data were analyzed using FlowJo software.

### Statistics

All measurements for ROS and 8-oxodG levels were performed in triplicate, and error bars on graphs represent standard deviations. The F-test was used to determine whether the variances between two variables were statistically significant. Statistical significances were analyzed using the Student’s *t* test and a p-value of < 0.05 was regarded as significant. Pearson’s correlation coefficient was used as a measure of linear correlation between two variables.

### Ethics approval and consent to participate

This research was determined by the Loma Linda University Institutional Review Board to not meet the definitions of human subject research, as no private individually identifiable information was obtained, there was no direct intervention or interaction, and only discarded tissue was used.

## Results

### Collection and preparation of cervical TZ and EC specimens

Cervical tissue includes the endocervix, the transformation zone, and the ectocervix. The endocervical mucosa is lined with a single layer of columnar mucous cells within the endocervical canal, while the ectocervix is covered with nonkeratinized stratified squamous epithelium directed towards the vaginal portion of the cervix^34^. The junction between these two types of epithelia is called the squamocolumnar junction (SCJ)^35^. In prepubertal girls, the functional SCJ is present within the endocervical canal. Upon entering puberty, hormonal influences cause the columnar epithelium to extend outwards over the ectocervix as the cervix everts, and also cause the SCJ to move outwards onto the vaginal portion of the cervix^35^. The zone of unstable epithelium between original SCJ and the new SCJ, which is internal to the original SCJ, is called the transformation zone (TZ)^35^. The TZ is a highly active metaplastic tissue in which the single layer columnar epithelium is transformed into the multilayered squamous epithelium of the ectocervix (EC). The cells of this zone, which are organized as single layer epithelium, are potential targets for HPV infection^36^. If these TZ cells are infected with HPVs, they can become the primary sites for cervical intraepithelial neoplasia development after replacement by non-keratinized stratified squamous epithelium^36^. Because these TZ cells are the likely targets for HPV, they were chosen for further investigation. The corresponding EC tissues were also analyzed, as they play a role in the virus life cycle.

Normal cervical tissues, isolated from patients undergoing vaginal hysterectomy, were dissected in cold PBS. The TZ and EC portions were separated, and specimens were prepared for measuring tissue ROS levels and for isolating primary keratinocytes for subsequent in vitro culturing. In total, specimens from 29 patients were collected. Twenty-nine TZ tissues and 22 EC tissues were analyzed for ROS levels, while the comparison of ROS levels between TZ and EC tissues was performed on specimens isolated from the common 22 patients. TZ tissue specimens isolated from the 29 patients were employed for isolation and culturing of primary keratinocytes from the TZ region.

The histology of the TZ and EC regions after dissection of a specimen are shown in Supplementary Fig. 2. The TZ region is characterized by single layer epithelium (Supplementary Fig. 2a), while the EC displays a morphology of multilayered squamous epithelium with basal/parabasal, intermediate and superficial layers (Supplementary Fig. 2b).

### ROS levels in cervical TZ tissues differ within a fivefold range between patients

**Figure 1:**
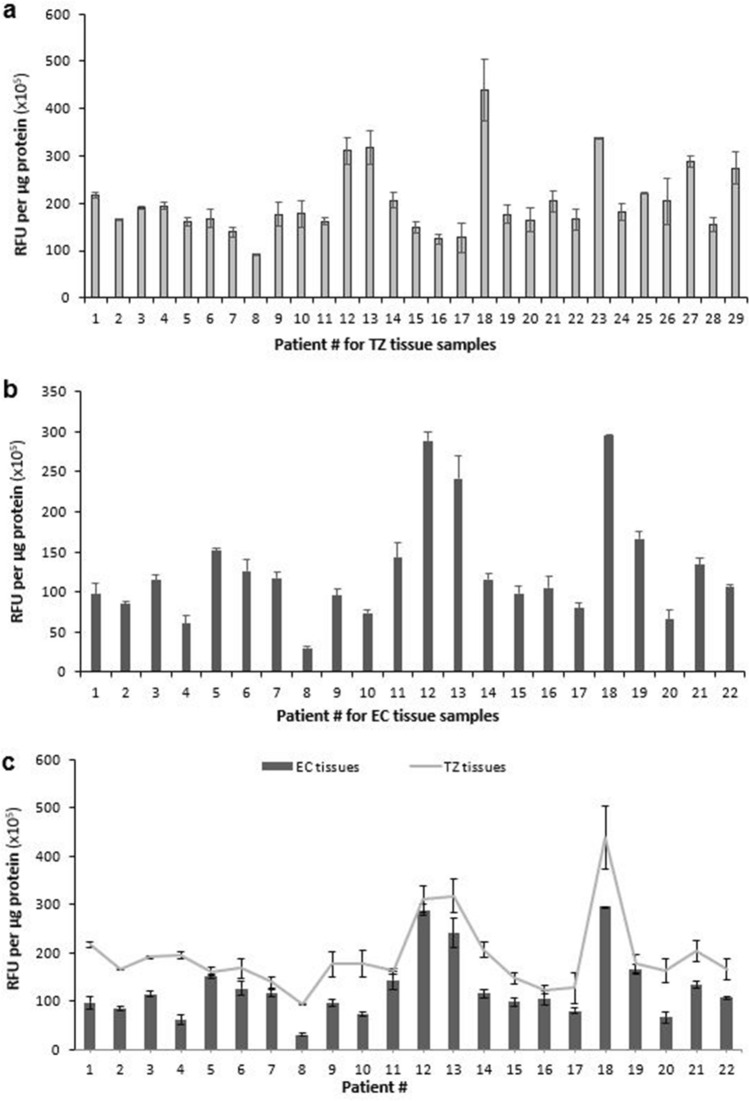
ROS levels in cervical TZ and corresponding EC tissues mirror each other and differ between patients within a range of five- and ten-fold, respectively. Homogenates were prepared from (**a**) TZ tissues and (**b**) EC tissues immediately after surgery, and ROS levels were assessed using the OxiSelect In Vitro ROS/RNS assay kit (Cell BioLabs) according to the manufacturer’s protocol. Protein concentrations were measured with the Coomassie Plus Assay Reagent (Thermo Scientific) and used for normalization. ROS levels are represented as relative fluorescence units (RFU)/µg protein. All measurements were performed in triplicate, and error bars represent the standard deviation. (**c**) ROS levels in TZ tissues and in the corresponding EC tissues (presented in **a**,**b**) are plotted on the same graph as line and bars, respectively. The F-test was used to determine whether the variances in ROS levels for TZ tissues and in ROS levels for EC tissues were significantly different.

The observation that OS is a promoting factor for numerous pathologies, including several types of cancer, suggested that an excess of free radicals could also contribute to HPV-induced cervical tumorigenesis. In particular, it seemed possible that differential background levels of ROS in cervical cells, caused by some combination of differences in genetic/epigenetic backgrounds together with variable exposure to exogenous factors, might contribute to the likelihood that a particular HPV-infected woman might or might not progress to cancer. To assess how the level of ROS in cervical tissues from different women might vary, TZ specimens isolated from 29 patients after vaginal hysterectomy were subjected to ROS analysis. The data obtained (Fig. 1a) demonstrate significant variability between patients in the level of ROS found in their TZ. For example, the lowest level of ROS was noted in sample #8 (93 × 10^5^ RFU/µg of protein), while the highest level was noted in samples #18 (438 × 10^5^ RFU/µg of protein). This represents a difference of about fivefold (*p* < 0.001). ROS levels for the other samples are distributed between these values.

### ROS levels in cervical EC tissues differ within a tenfold range between patients

TZ cells are the primary targets for HPV infection. However, after replacement with non-keratinized stratified squamous epithelium, the HPV life cycle takes place in the multilayered squamous epithelium of the EC. Therefore, we also measured the levels of ROS in EC tissues isolated from 22 patients following vaginal hysterectomy. Our data in Fig. 1b shows that the levels of ROS are also highly variable between the different EC samples, having a tenfold difference (*p* < 0.001) between the lowest and highest samples. Similar to the TZ tissues, sample #8 (30 × 10^5^ RFU/µg of protein) displayed the lowest level of ROS, while the highest levels of ROS in EC tissues were reported in samples #12 (289 × 10^5^ RFU/µg of protein) and #18 (296 × 10^5^ RFU/µg of protein). The ROS level variability observed in both TZ and EC regions are likely to reflect contributions from genetic/epigenetic regulation of ROS levels, together with influences from exogenously derived factors such as exposure to drugs, environmental factors and lifestyle.

### ROS levels in TZ tissues correspond to those observed in their corresponding EC tissues

As described above, the EC and TZ tissues differ both functionally and structurally. We therefore compared the detected levels of ROS between the single- and multilayered epithelium from the cervical specimens of the common 22 patients. Figure 1c represents the levels of ROS in EC tissues (in bars), as well as those of their corresponding TZ tissues (in a line plot). Despite the greater variability in EC compared to TZ tissues, likely due to differences in tissue composition, the overall pattern of ROS levels in TZ tissues mirrored those obtained in their corresponding EC tissues with a Pearson’s correlation coefficient of r = 0.85 (*p* < 0.001). These results reveal a good correlation between ROS levels in TZ tissues and their corresponding EC tissues. To determine whether the variances in ROS levels for TZ tissues and for EC tissues were significantly different, we employed the F-test. The F-value was equal to 1.23, a value that is lower than F-critical (2.05), thereby demonstrating that the two variables do not differ significantly and that the difference between values could be explained by random events. Interestingly, within each pair (TZ and EC), the levels of ROS in the TZ was always either higher than or approximately equal to that observed in the EC homogenate. For example, in samples #4, #8, #10, and #20, the difference between the TZ and EC ROS levels was more than 2.5-fold (*p* < 0.01), while in samples #5, #12, #16, and #19, there was no significant difference between the two.

### Culture of primary keratinocytes from TZ specimens

Dissection of the primary tissue enables us to obtain approximately 200 mg of TZ or EC tissues, which consists of a keratinocyte layer, along with fat and connective tissues. This amount of material (a few mm^3^ in volume) is sufficient for only a limited number of studies (namely histological examination and ROS measurement). Therefore, in order to increase the amount of material available for investigations focused on areas such as the modeling of HPV infection, HPV integration and cellular transformation, we applied known procedures to isolate and culture primary keratinocytes from the TZ cervical tissues. One property that frequently restricts keratinocyte production is the well-known fact that primary keratinocytes in culture undergo terminal differentiation after several divisions. We were able to overcome this restriction and create a culture that can divide indefinitely by blocking the ROCK pathway using the Y-27632 inhibitor of serine/threonine kinases ROCK-I and ROCK-II^37^. Application of ROCK inhibitors to keratinocytes has been shown to prevent terminal differentiation and induce cell division^37^. Using Y-27632, we were able to maintain the keratinocyte culture for more than 6 months without noticeable changes in the growth rate or morphology. The morphology of our TZ cervical keratinocytes grown in culture is shown in Supplementary Fig. 3.

### Variability of ROS levels in cultured primary cervical keratinocytes

**Figure 2:**
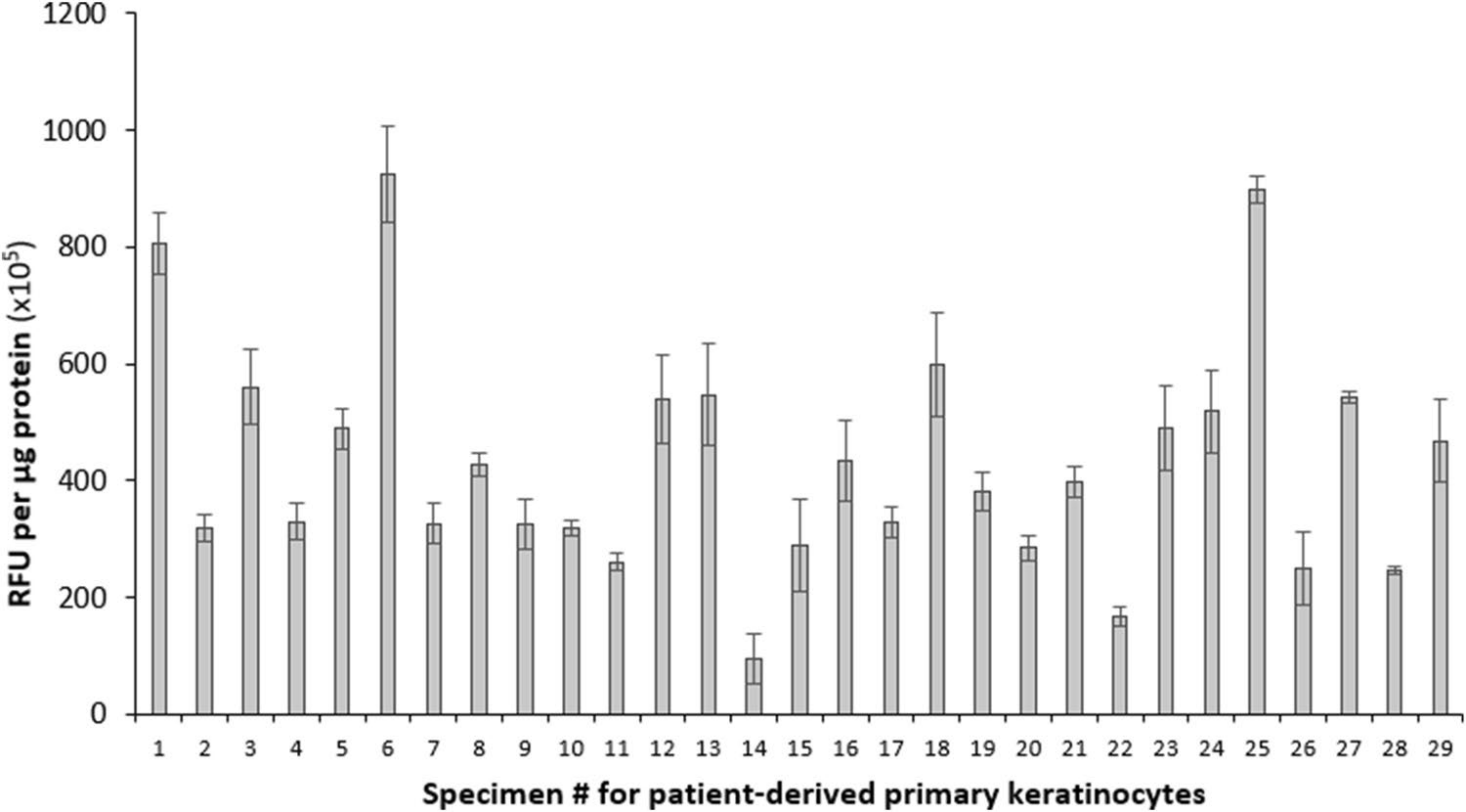
ROS levels in primary keratinocytes isolated from TZ specimens are variable within a range of about ten-fold between patients. 2.5 × 10^6^ primary cells were homogenized and assessed for ROS levels using the OxiSelect In Vitro ROS/RNS assay kit (Cell BioLabs) according to the manufacturer’s protocol. Protein concentrations were measured with the Coomassie Plus Assay Reagent (Thermo Scientific) and used for normalization. ROS levels are represented as relative fluorescence units (RFU)/µg protein. All measurements were performed in triplicate, and error bars represent the standard deviation.

To assess how closely our cultured primary keratinocytes reflected the levels of ROS observed in their corresponding tissues of origin, we measured the levels of ROS in the primary keratinocytes isolated from the TZ regions of 29 patients and compared them to those detected in their corresponding tissues. The levels of ROS in the 29 cultured patient-derived primary keratinocytes are represented in Fig. 2. Our data show that the ROS levels are highly variable between TZ keratinocytes isolated from different patients. The lowest level of ROS was found in the TZ keratinocytes isolated from patient #14 (96 × 10^5^ RFU/μg protein), while the highest values were detected in patient #6 (926 × 10^5^ RFU/μg protein) and patient #25 (899 × 10^5^ RFU/μg protein). This represents a difference of approximately tenfold (*p* < 0.001).

**Figure 3:**
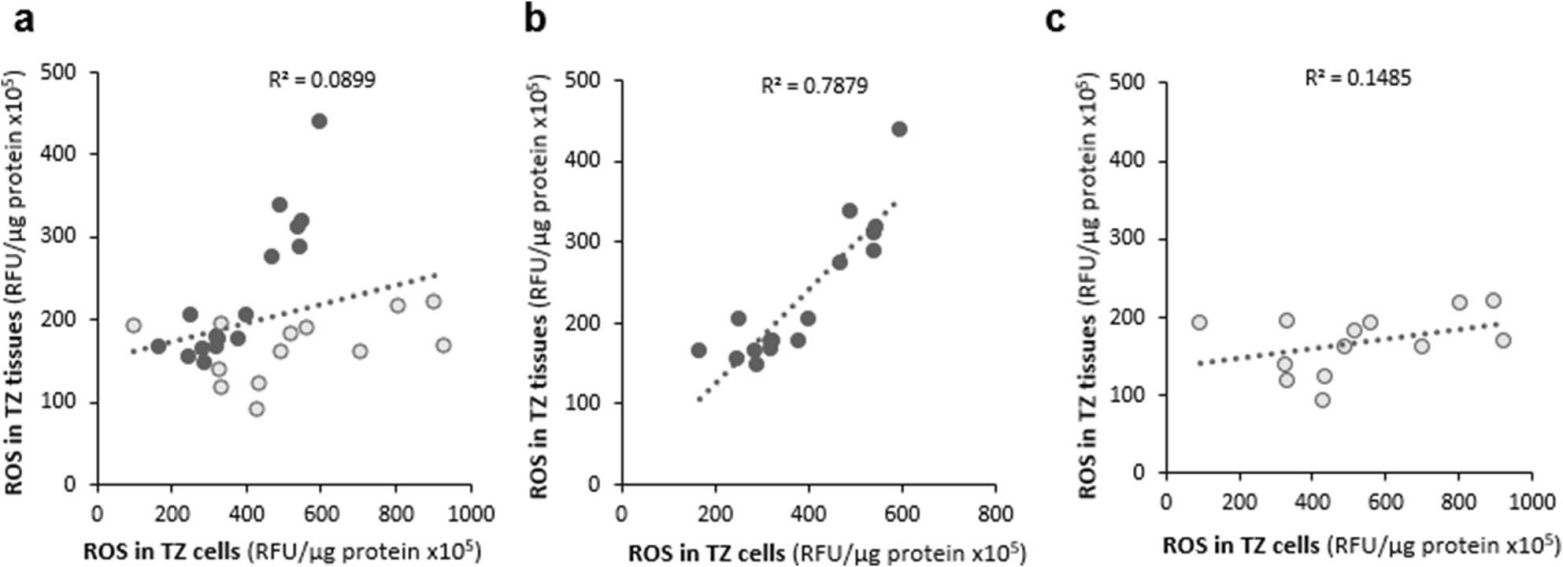
The levels of ROS in cultured keratinocytes mirror those observed in the corresponding cervical tissue specimens in some but not all patients. (**a**) Scatterplot displaying the levels of ROS in TZ tissues vs the corresponding cultured keratinocytes in all patients (n = 29). (**b**) Scatterplot displaying the levels of ROS in cultured keratinocytes strongly mirroring ROS levels in the corresponding TZ tissues (n = 16). (**c**) Scatterplot displaying the levels of ROS in cultured keratinocytes weakly mirroring ROS levels in the corresponding TZ tissues (n = 13).

**Figure 4:**
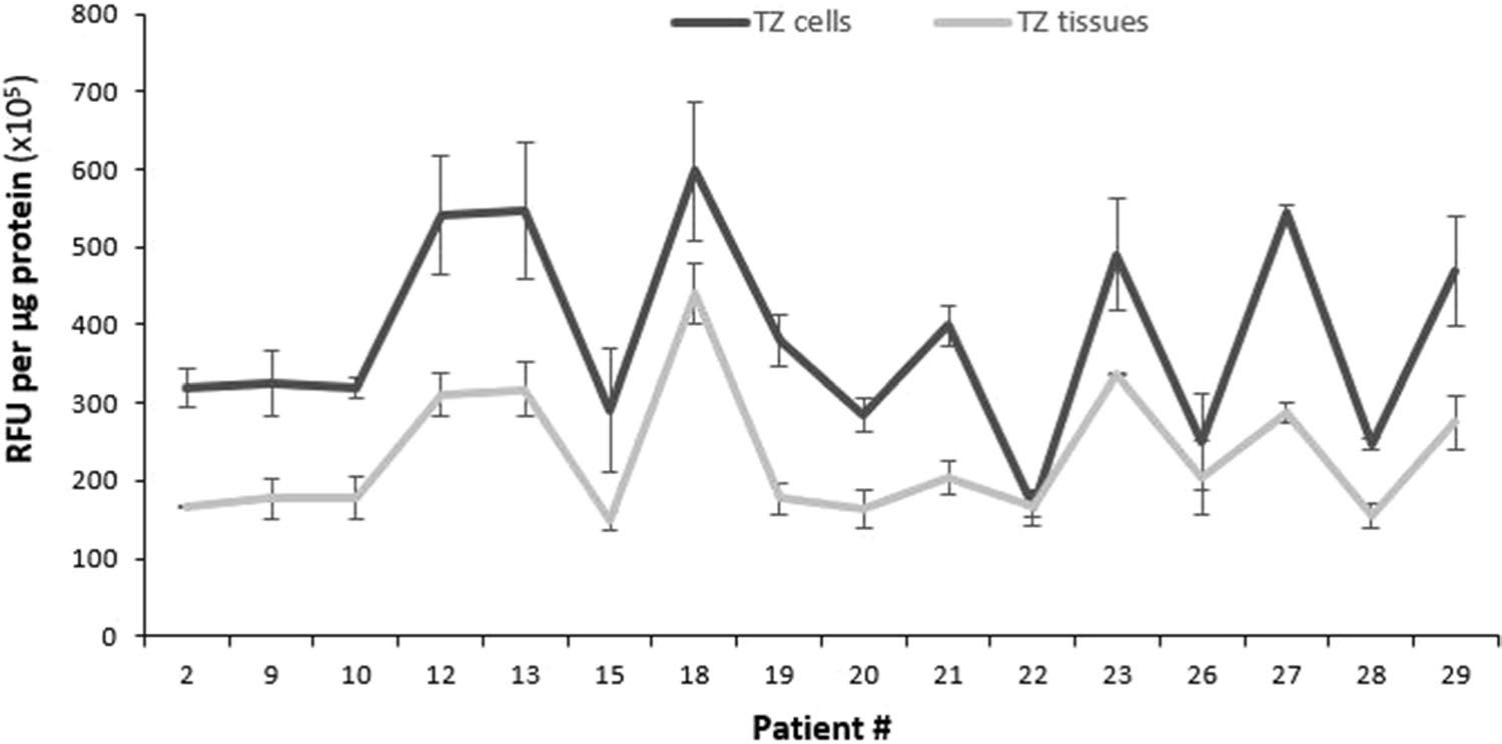
The levels of ROS in cultured keratinocytes strongly mirror those observed in the corresponding TZ tissues in 16 patients. The dataset of the patients that displayed a strong correlation of ROS levels in cultured keratinocytes vs their corresponding tissues (shown in Fig. 3b) is presented in a line graph.

Surprisingly, when we compared the levels of ROS detected in tissues (Fig. 1a) with those observed in cultured keratinocytes isolated from those same tissues (Fig. 2), an R^2^ = 0.0899 was detected, signifying that only about 9% of ROS levels in the tissue homogenates corresponded to those obtained in their respective cultured keratinocytes (Fig. 3a). However, the scatterplot displays two distinct populations (represented by 2 different colors): the first population (in dark gray) contains samples that display a strong positive linear association between ROS levels in cultured keratinocytes vs their corresponding tissues with a Pearson’s correlation coefficient of r = 0.888 (*p* < 0.001) (Fig. 3b), while the other population (in light gray) includes samples that have a much weaker correlation with a Pearson’s correlation coefficient of only r = 0.385 (ns) (Fig. 3c). The pattern of ROS levels in the tissue samples strongly mirroring that observed in the corresponding primary keratinocytes is displayed in Fig. 4. Because the keratinocytes were isolated, cultured and grown in vitro conditions, the trends seen in the isolated primary keratinocytes exclusively point toward biological factors such as the cell’s genetic and epigenetic background, thereby enabling us to dissect out genetic/epigenetic influences from the environmental factors that are represented in the observations from tissue samples.

### High levels of 8-oxodG, a marker of DNA damage, correlate with higher levels of ROS in cultured keratinocytes

**Figure 5:**
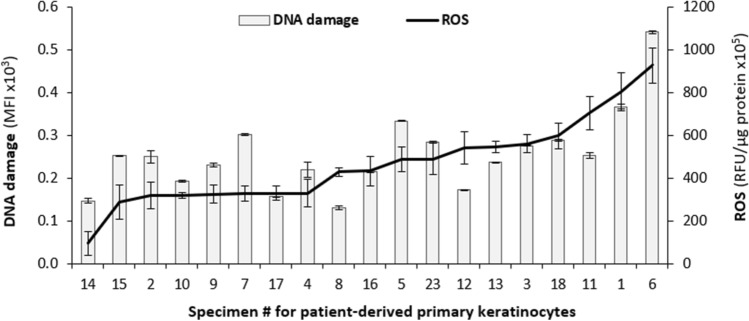
The levels of DNA damage approximately mirror the observed ROS levels in the cervical keratinocytes. DNA damage was assessed by detection of 8-oxodG using Avidin-FITC. Cells were fixed in 4% paraformaldehyde, permeabilized with ethanol, and stained with 2 µg/ml of Avidin-FITC for 1 h. The level of bound Avidin-FITC, presented as mean fluorescence intensity (MFI (Avidin-FITC)) was detected by flow cytometry and analyzed using FlowJo software. The MFI values for DNA damage (Avidin-FITC staining) were plotted as bars on a graph displaying the levels of ROS in an increasing order as a line plot. To determine whether the variances in ROS levels and in 8-oxodG levels were significantly different, the F-test was used.

One major outcome of higher levels of cellular ROS is an induction of chronic OS. OS is well known for its ability to damage multiple biomolecules, including DNA, and the oxidation-induced incorporation of 8-oxodG into DNA lesions can lead to mismatch mutations during DNA synthesis^15,24,38^. The data shown in Fig. 2 demonstrates that ROS levels differ between primary keratinocytes derived from different patients. The well-established linkage between OS and DNA damage therefore suggested that cells with higher levels of ROS would also display higher level of 8-oxodG. To test this idea, we measured the level of 8-oxodG in 19 patient-derived cultured keratinocytes that displayed variable levels of ROS. 8-oxodG levels were estimated by flow cytometry following Avidin-FITC staining, as avidin is able to bind to lesions containing 8-oxodG^39^. Figure 5 represents the measured levels of 8-oxodG in a graph displaying the levels of ROS in an increasing order in the 19 patient-derived keratinocyte lines. Our results show that the levels of DNA damage approximately mirror the levels of ROS in these patient-derived cells. For instance, the highest levels of ROS were detected in TZ keratinocytes isolated from specimens #1 and #6; these cells are also the ones displaying the highest levels of 8-oxodG. The other specimens also displayed good correspondence between their levels of ROS and 8-oxodG. These results revealed a positive linear correlation between ROS levels and DNA damage in the cervix with a Pearson’s correlation coefficient of r = 0.72, representing a strong relationship between the 2 variables *(p* < 0.001). These data, therefore, are consistent with the idea that higher levels of cellular oxidative stress, as determined by increased levels of ROS, cause a downstream increase in the level of DNA damage.

### Contribution of ethnicity and age to ROS level variability.

Cervical cancer incidence displays a significant health disparity, with Hispanic women having nearly double the incidence rate of non-Hispanic white women^40–42^. In addition, one of the risk factors associated with cervical cancer is the age of HPV-infected women. We hypothesized that oxidative stress levels in various populations of different races and ages vary, and have the potential to influence the observed health disparity regarding cervical cancer incidence. We therefore analyzed the contribution of such factors to the ROS level variabilities observed in the cervical tissues and cells.

**Figure 6:**
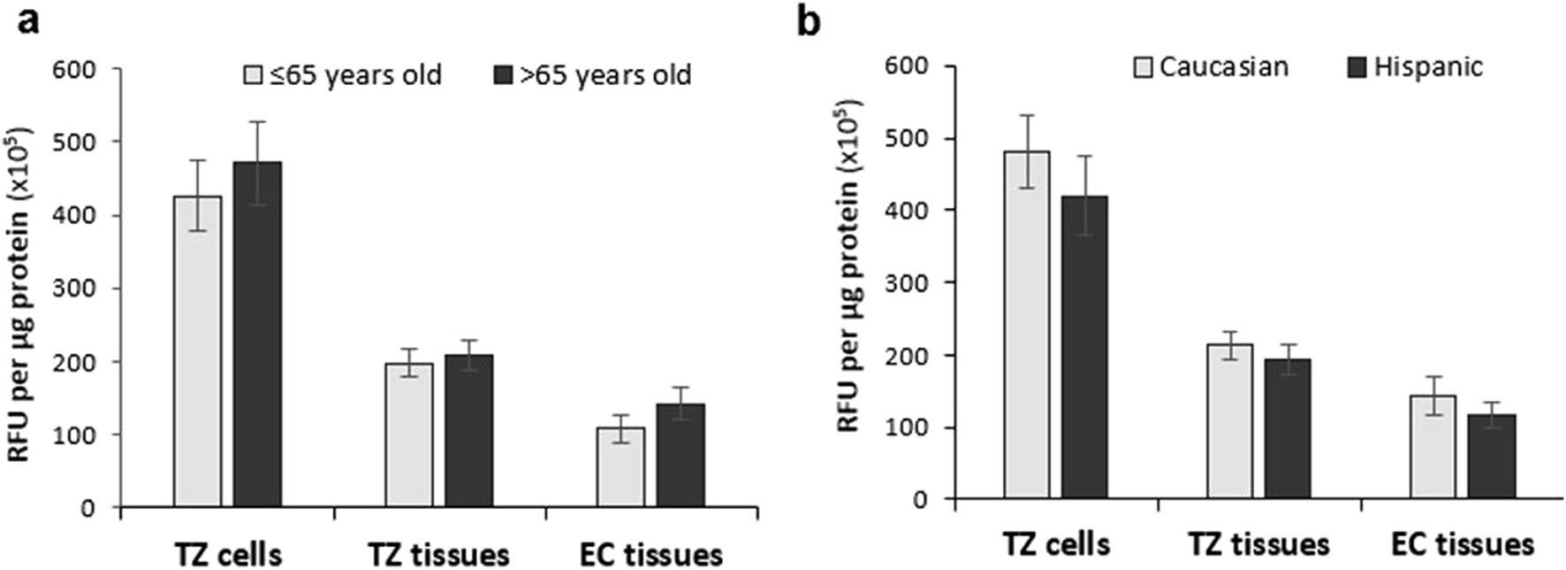
Contribution of patients’ age and ethnicity to the observed ROS level variabilities in the cervical specimens. (**a**) The patients from which the cervical specimens were collected ranged between 35 and 84 years old (with a median age of 65) and therefore were grouped into two categories: < 65 years vs ≥ 65 years old. (**b**) Patients are either classified as Caucasian or Hispanic. The F-test was used to determine whether the variances in ROS levels between the 2 groups were significantly different.

The patients from which the cervical specimens were collected ranged between 35 and 84 years old (with a median age of 65) and were classified as either Caucasian or Hispanic (Supplementary Table 1). Figure 6a shows that women older than 65 years old had relatively higher ROS levels, compared to those with an age of ≤ 65 years old, in both TZ and EC tissues as well as cultured TZ cells, consistent with reports of higher risk of progression to cervical cancer in older HPV-infected women. On the other hand, Caucasian women had moderately higher ROS levels than Hispanics in their cervical tissues and cells (Fig. 6b), despite higher incidence of cervical cancer in Hispanic women. The results were not statistically significant, likely due to the limited number of samples in which ROS levels were measured.

## Discussion

In this report, we demonstrate for the first time a significant variability between women with regards to the background ROS levels found in both their cervical TZ and EC tissues (Fig. 1a,b). In particular, the difference between the lowest and highest levels of ROS species in TZ and EC tissues was approximately fivefold and tenfold, respectively. Despite the greater variability in EC compared to TZ tissues, likely due to differences in tissue composition, the overall pattern of ROS levels in TZ tissues corresponded to that obtained in their corresponding EC tissues (Fig. 1c). A comparison of ROS levels between single- (TZ) and multilayered (EC) epithelium from cervix has also not been previously reported. Interestingly, we found that the levels of ROS in TZ tissues were always either higher than or equal to the ROS levels found in the respective EC tissues. This difference may suggest a reason that TZ tissue is the primary target for HPV infection and cervical cancer development. It is generally assumed that the TZ epithelium is the site of neoplastic changes, and the most common area for the origination of cervical cancer^36,43^. The formation of cervical lesions may be facilitated by HPV infection of TZ cells, which can subsequently go on to form the basal layer of the stratified epithelium of EC where HPV life cycle occurs.

Epidemiological and molecular data both point toward OS as an important contributor to cervical cancer development. For example, epidemiological data link conditions known to cause OS and DNA damage, such as smoking and co-infection with the STD-associated pathogens *Chlamydia trachomatis* and *Neisseria gonorrhoeae*, with increased incidence of HPV-mediated cancers (reviewed in^21,22^). It has also been reported that greater oxidative DNA damage exists in HPV-related dysplastic cervical lesions compared to samples with normal cytology, especially in women with high-grade squamous intraepithelial lesions^44^. In addition to the exogenously-derived risk factors discussed above, Munoz et al.^18^ have suggested that host-related factors, such as endogenous hormone levels, genetic background and factors related to the immune response, could also contribute to cancer promotion. Logic for this suggestion comes from the fact that under normal circumstances, ROS levels are maintained in a condition of homeostasis by balanced functioning of pro- and anti-oxidant systems^26,27^ that are regulated by individual genetic determinants. Besides such genetic determinants, life-style and physiological factors can also influence gene expression levels through changes to epigenetic regulation^45^. Both genetic and epigenetic factors are examples of biological factors that can contribute to the variability in levels of ROS observed in different individuals.

The evidence presented in this report indicates an important role for individual genetic and epigenetic variation in determining the level of cellular ROS and importantly, the resulting level of DNA damage. For the first time, we have demonstrated that individual women display significantly different levels of ROS and DNA damage in their cervical cells (Fig. 5). Furthermore, the levels of ROS correlate with those of DNA damage. The higher levels of ROS seen in some women may reflect variability in alleles, epigenetic regulation and/or mutation rates.

The results reported here also begin to address the question of the relative roles played by environmental agents and other external factors *vs* internal factors such as genetic variation and epigenetic programming. While acute OS induced by exogenously derived factors can, in principle, be eliminated by removing the insult, the effect of cellular OS exerted by biological factors represents a more chronic and constant influence. The variability of ROS levels in cervical tissues observed in different women (Fig. 1a,b) could, in principle, be mediated both by exogenous factors and by genetic/epigenetic factors that control the level of ROS, as the genetic and epigenetic capacity of cells to deal with oxidative stress will affect the ability of those cells to deal with both endogenous and exogenous insults. In contrast, the cultured keratinocytes from different individuals represent a model system in which variability in the influence of exogenous factors is removed because the cells are maintained in the same in vitro conditions for an extended period. Interestingly, the comparison between cervical tissues and the corresponding cultured keratinocytes (Figs. 1a and 2) in the total population of samples revealed a strong variability in ROS levels with a coefficient of determination of only 0.0899 (Fig. 3a). However, the scatterplot displays two distinct populations (represented in 2 different colors): Some of the specimens display a strong correlation of ROS levels in their tissue homogenates vs cultured cells with a Pearson’s correlation coefficient of r = 0.888 (*p* < 0.001) (Fig. 3b), indicating that ROS levels in their tissues are likely determined primarily by biological factors of the patients. On the other hand, the rest of the samples display significant variability in the trend of ROS levels between tissue and cell homogenates (Fig. 3c), suggesting that ROS levels in the tissue samples are likely determined primarily by exogenous/environmental factors in these patients. This finding is important for future studies, as it enables us to begin dissecting out genetic/epigenetic influences from environmental influences, noting that trends seen in the isolated keratinocytes will primarily point toward genetic/epigenetic contributions. One potential influential factor that needs to be examined in the future is the hormonal status of the women. Parity and use of oral contraceptives, both of which are associated with increased circulating levels of sex hormones, are classified as co-factors that predispose HPV-infected women to cervical cancer^46–48^. It has been demonstrated that long-term use of oral contraceptives increases the risk of cervical cancer by up to four-fold in HPV-infected women^49^. Similarly, the hormonal changes along with the cervical trauma during pregnancy increase the odds ratio for cervical cancer to 3.8 after 7 full-term pregnancies compared with nulliparous women, and to 2.3 compared to women with 1 or 2 full-term pregnancies^50^. Relevant to our study, the impact of these hormonal factors on the risk of developing cervical cancer may be mediated via an increased oxidative stress status in the host cells, since the female steroid estrogens and their various metabolites stimulate ROS production to activate various cell signaling pathways^51–56^. Defining the molecular mechanisms that contribute to differential levels of ROS and DNA damage in different women will provide direction toward inquiries designed to understand why the disease is more prevalent in certain populations than in others.

Within the US, cervical cancer manifests as a major health disparity between different ethnic groups. Hispanic women have one of the highest cervical cancer incidence rates of any racial/ethnic group in the United States; nearly double that of non-Hispanic white women^40–42^. Eliminating this health disparity requires an appreciation of how factors related to race and ethnicity may influence the process through which cervical cancer develops. A significantly different infection rate is unlikely to be the factor most responsible for the observed health disparity, because it is estimated that most sexually active men and women of all races and ethnicities have been infected with HPV at some point in their lives^57,58^. This suggests that the health disparity associated with cervical cancer presents downstream of infection and upstream of cancer. Abundant literature reports indicate that race/ethnicity-related differences can contribute to differences in the background levels of ROS^59,60^, and host genetic backgrounds have been shown to influence the incidence of cervical cancer in Sweden^61^, the eastern US and Costa Rica^62^. However, we failed to find statistically significant differences in the levels of ROS between the cervical specimens of our two groups (Hispanic vs Caucasian) (Fig. 6b). While this could be due to the limited number of samples analyzed, our preliminary data suggest that the racial/ethnic disparity in cancer incidence may be primarily tied to factors such as socio-economic status and access to healthcare^63^ rather than to genetic/epigenetic differences. In fact, significant differences exist between racial/ethnic groups with regards to vaccination^64^ and pap screening^65^, indicating that efforts to reduce this disparity should focus on such issues.

Finally, we investigated the contribution of age to the ROS level variability observed in the cervical specimens in order to explore its potential influence on cervical cancer development. Age is a known risk factor for cervical cancer. The disease tends to occur in midlife and is most frequently diagnosed in women between the ages of 35 and 44. However, the risk of developing cervical cancer is still present as women get older and does not decline until age ≥ 85 years old^66,67^. Furthermore, it is well established that oxidative stress and aging are closely connected since age-associated functional losses are mainly due to the progressive accumulation of ROS-induced damages. In fact, oxidative stress is reported to be involved in several age-related conditions such as cardiocascular diseases, chronic obstructive pulmonary disease, neurodegenerative diseases and cancer^68,69^. Our data suggest that women with an age of > 65 years old displayed relatively higher levels of ROS in their cervical specimens compared to those with an age of ≤ 65 years (Fig. 6a). While this data is not statistically significant due to the limited amount of samples analyzed, it is consistent with reports of higher risk of progression to cancer in older HPV-infected women.

The importance of our observations may lie in the possible clinical consequences of higher levels of ROS in the cervices of some women, and in particular, how these higher levels of ROS may affect the integration of HPV. Integration of HPV genome into that of the host is considered to function as a critical step in the development of most cases of cervical cancer. In ~ 80–90% of specimens obtained from cervical cancers, HPV DNA is found integrated into the host genomes^70–73^. In many of the cases, the mechanism through which HPV integration leads to cancer development is thought to be a loss of functional E2 (a negative regulator of E6 and E7 expression) due to linearization, followed by increased expression of the E6 and E7 oncogenes. The resulting over-expression of E6 and E7 increases cellular proliferation at the same time that it decreases responsiveness to apoptotic signals, leading to cancer development.

Integration proceeds by way of non-homologous recombination, and therefore requires linearization of the viral episome and breakage of the host chromosome. Recently, we demonstrated that depletion of the antioxidant glutathione induced oxidative DNA damage and led to an increase in the frequency of HPV integration in human cervical keratinocytes that contained episomal HPV^74^. Consistent with these proposed connections between OS, DNA damage, HPV integration and cancer, integration associated with oxidative stress has also been demonstrated for another DNA virus, Hepatitis B^75–77^. Virus-derived factors, such as co-infection with other viruses, viral load and persistence of HPV infection can also promote HPV integration^18^. These virus-associated factors may also be connected to increased OS; recently, we demonstrated that expression of the E6 splice variant, E6*, but not the full-length version of E6, induced increases in ROS, DNA damage^78^ and in the frequency of foreign DNA integration^74^. Importantly, our current study suggests that if women exhibiting high oxidative stress were to be harboring episomal HPV DNA, an increased probability of integration could result, explaining why some, but not all, HPV-infected women develop cervical cancer. Furthermore, high levels of ROS in TZ cells infected with HPV have the potential to increase expression of the E6/E7 oncogenes^79^, leading to higher levels of DNA damage^80^, and inhibit pro- and repress anti-tumor pathways^81^. It is well known that OS itself is a tumor-promoting factor; in cooperation with HPV, it becomes a more potent carcinogen.

Overall, our findings describe significant variability in ROS levels among women and shed light on the potential contribution of increased levels of ROS, and the exogenous/environmental and genetic/epigenetic contexts that support these increased levels, to HPV-mediated carcinogenesis. Future research will focus on exploring the possibility that high levels of ROS may predispose certain infected women and populations to HPV-mediated carcinogenesis. Our overall goal is to develop an understanding of the underlying mechanisms so as to develop novel and effective ways to intercept cancer development in HPV-infected individuals.

## Conclusions

We characterized normal, non-cancerous cervical tissues for their levels of ROS, and demonstrated five and ten-fold variability in the levels of ROS between different TZ and EC tissues, respectively. Despite the greater variability in EC compared to TZ tissues, likely due to differences in tissue composition, the overall pattern of ROS levels in TZ tissues mirrored those obtained in their corresponding EC tissues. Our results also show that the levels of ROS in TZ tissues were higher than or equal to the ROS levels found in the respective EC tissues, providing an explanation for TZ tissue being the primary target for HPV infection and cervical cancer development. Interestingly, primary keratinocytes isolated and cultured from the cervical specimens also displayed high variability in ROS levels, with some strongly mirroring the levels of ROS observed in their corresponding tissues, while others displayed a much weaker association. These results allow us to begin distinguishing the environmental influences from the genetic/epigenetic background of the patients. Finally, we were able to demonstrate that the level of DNA damage mirrors the level of ROS in the cultured primary cells.

## Supplementary information

Supplementary Information1

## Data Availability

The datasets used and/or analyzed during the current study are available from the corresponding author on reasonable request.

## References

[1] Bernard W, Stewart CPW (2014). Cancers of the female reproductive organs. World Cancer Rep..

[2] Bray F (2018). Global cancer statistics 2018 GLOBOCAN estimates of incidence and mortality worldwide for 36 cancers in 185 countries. CA Cancer J. Clin..

[3] Ahn WS (2003). Evaluation of adenoassociated virus 2 and human papilloma virus 16 and 18 infection in cervical cancer biopsies. Gynecol. Oncol..

[4] Muñoz N (2003). Epidemiologic classification of human papillomavirus types associated with cervical cancer. N. Engl. J. Med..

[5] Smith JS (2007). Human papillomavirus type distribution in invasive cervical cancer and high-grade cervical lesions: A meta-analysis update. Int. J. Cancer.

[6] Franco EL (1999). Epidemiology of acquisition and clearance of cervical human papillomavirus infection in women from a high-risk area for cervical cancer. J. Infect. Dis..

[7] Molano M (2003). Determinants of clearance of human papillomavirus infections in Colombian women with normal cytology: A population-based, 5-year follow-up study. Am. J. Epidemiol..

[8] Braaten KP, Laufer MR (2008). Human papillomavirus (HPV), HPV-related disease, and the HPV vaccine. Rev. Obstetr. Gynecol..

[9] Banik U (2011). Pattern of epithelial cell abnormality in Pap smear: A clinicopathological and demographic correlation. Cytojournal.

[10] Haverkos HW (2005). Multifactorial etiology of cervical cancer: A hypothesis. MedGenMed.

[11] De Marco F (2012). Oxidative stress in HPV-driven viral carcinogenesis: Redox proteomics analysis of HPV-16 dysplastic and neoplastic tissues. PLoS ONE.

[12] De Marco F (2013). Oxidative stress and HPV carcinogenesis. Viruses.

[13] Waris G, Ahsan H (2006). Reactive oxygen species: Role in the development of cancer and various chronic conditions. J. Carcinog..

[14] Halliwell B (2007). Oxidative stress and cancer: Have we moved forward?. Biochem. J..

[15] Cooke MS (2003). Oxidative DNA damage: Mechanisms, mutation, and disease. FASEB J..

[16] Haverkos HW (2003). Cigarette smoking and cervical cancer: Part I: a meta-analysis. Biomed. Pharmacother..

[17] Haverkos H, Rohrer M, Pickworth W (2000). The cause of invasive cervical cancer could be multifactorial. Biomed. Pharmacother..

[18] Munoz N (2006). Chapter 1: HPV in the etiology of human cancer. Vaccine.

[19] Plummer M (2003). Smoking and cervical cancer: Pooled analysis of the IARC multi-centric case–control study. Cancer Causes Control.

[20] Tollefson AK (2010). Endogenous enzymes (NOX and ECSOD) regulate smoke-induced oxidative stress. Free Radic. Biol. Med..

[21] Williams VM (2011). HPV-DNA integration and carcinogenesis: Putative roles for inflammation and oxidative stress. Fut. Virol..

[22] Chen Y (2014). Viral carcinogenesis: Factors inducing DNA damage and virus integration. Cancers (Basel).

[23] Klaunig JE, Kamendulis LM, Hocevar BA (2010). Oxidative stress and oxidative damage in carcinogenesis. Toxicol. Pathol..

[24] Olinski R (1992). DNA base modifications in chromatin of human cancerous tissues. FEBS Lett..

[25] Zienolddiny S, Ryberg D, Haugen A (2000). Induction of microsatellite mutations by oxidative agents in human lung cancer cell lines. Carcinogenesis.

[26] Ray PD, Huang BW, Tsuji Y (2012). Reactive oxygen species (ROS) homeostasis and redox regulation in cellular signaling. Cell Signal.

[27] D'Autreaux B, Toledano MB (2007). ROS as signalling molecules: Mechanisms that generate specificity in ROS homeostasis. Nat. Rev. Mol. Cell Biol..

[28] Salganik RI (2001). The benefits and hazards of antioxidants: Controlling apoptosis and other protective mechanisms in cancer patients and the human population. J. Am. Coll. Nutr..

[29] Agarwal A, Saleh RA, Bedaiwy MA (2003). Role of reactive oxygen species in the pathophysiology of human reproduction. Fertil. Steril..

[30] Dato S (2013). Exploring the role of genetic variability and lifestyle in oxidative stress response for healthy aging and longevity. Int. J. Mol. Sci..

[31] Zhang X (2014). Genetic variants and risk of cervical cancer: Epidemiological evidence, meta-analysis and research review. BJOG.

[32] Lynch HT (1998). Hereditary factors in gynecologic cancer. Oncologist.

[33] Magnusson PK, Gyllensten UB (2000). Cervical cancer risk: Is there a genetic component?. Mol. Med. Today.

[34] Gray H, Williams PL, Bannister LH (1995). Gray's anatomy: the anatomical basis of medicine and surgery.

[35] Beckmann CRB, American College of Obstetricians and Gynecologists (2014). Obstetrics and gynecology.

[36] Schiffman M (2007). Human papillomavirus and cervical cancer. Lancet.

[37] McMullan R (2003). Keratinocyte differentiation is regulated by the Rho and ROCK signaling pathway. Curr. Biol..

[38] Katerji M, Filippova M, Duerksen-Hughes P (2019). Approaches and methods to measure oxidative stress in clinical samples: Research applications in the cancer field. Oxidat. Med. Cell. Longev..

[39] Achanta G, Huang P (2004). Role of p53 in sensing oxidative DNA damage in response to reactive oxygen species-generating agents. Cancer Res..

[40] Mann L (2015). Increasing cervical cancer screening among US hispanics/latinas: A qualitative systematic review. J. Cancer Educ..

[41] Khan HMR (2016). Disparities in cervical cancer characteristics and survival between white hispanics and white non-hispanic women. J. Women's Health.

[42] Yu L, Sabatino SA, White MC (2019). Rural–Urban and racial/ethnic disparities in invasive cervical cancer incidence in the United States, 2010–2014. Prev. Chron. Dis..

[43] Herfs M (2012). A discrete population of squamocolumnar junction cells implicated in the pathogenesis of cervical cancer. Proc. Natl. Acad. Sci. U.S.A..

[44] Visalli G (2016). Higher levels of oxidative DNA damage in cervical cells are correlated with the grade of dysplasia and HPV infection. J. Med. Virol..

[45] Jaenisch R, Bird A (2003). Epigenetic regulation of gene expression: How the genome integrates intrinsic and environmental signals. Nat. Genet..

[46] Shields TS (2004). A case–control study of endogenous hormones and cervical cancer. Br. J. Cancer.

[47] Roura E (2016). The influence of hormonal factors on the risk of developing cervical cancer and pre-cancer: Results from the EPIC cohort. PLoS ONE.

[48] de Villiers E-M (2003). Relationship between steroid hormone contraceptives and HPV, cervical intraepithelial neoplasia and cervical carcinoma. Int. J. Cancer.

[49] Moreno V (2002). Effect of oral contraceptives on risk of cervical cancer in women with human papillomavirus infection: The IARC multicentric case-control study. Lancet.

[50] Muñoz N (2002). Role of parity and human papillomavirus in cervical cancer: The IARC multicentric case–control study. Lancet.

[51] Mobley JA, Brueggemeier RW (2004). Estrogen receptor-mediated regulation of oxidative stress and DNA damage in breast cancer. Carcinogenesis.

[52] Roy D (2007). Estrogen-induced generation of reactive oxygen and nitrogen species, gene damage, and estrogen-dependent cancers. J. Toxicol. Environ. Health B Crit. Rev..

[53] Fussell KC (2011). Catechol metabolites of endogenous estrogens induce redox cycling and generate reactive oxygen species in breast epithelial cells. Carcinogenesis.

[54] Felty Q (2005). Estrogen-induced mitochondrial reactive oxygen species as signal-transducing messengers. Biochemistry.

[55] Bhat HK, Patel MM (2004). Differential oxidant potential of carcinogenic and weakly carcinogenic estrogens: Involvement of metabolic activation and cytochrome P450. Can. Res..

[56] Okoh V, Deoraj A, Roy D (2011). Estrogen-induced reactive oxygen species-mediated signalings contribute to breast cancer. Biochim. Biophys. Acta.

[57] Cates W (1999). Estimates of the incidence and prevalence of sexually transmitted diseases in the United States. American Social Health Association Panel. Sex Transm. Dis..

[58] Koutsky L (1997). Epidemiology of genital human papillomavirus infection. Am. J. Med..

[59] Morris AA (2012). Differences in systemic oxidative stress based on race and the metabolic syndrome: The Morehouse and Emory Team up to Eliminate Health Disparities (META-Health) study. Metab. Syndr. Relat. Disord..

[60] Feairheller DL (2011). Racial differences in oxidative stress and inflammation: IN vitro and in vivo. Clin. Transl. Sci..

[61] Czene K (2002). P Lichtenstein, K Hemminki, Environmental and heritable causes of cancer among 9.6 million individuals in the Swedish family-cancer database. Int. J. Cancer.

[62] Zelmanowicz ADM (2005). Family history as a co-factor for adenocarcinoma and squamous cell carcinoma of the uterine cervix: Results from two studies conducted in Costa Rica and the United States. Int. J. Cancer.

[63] Hicks ML (2006). Disparities in cervical cancer screening, treatment and outcomes. Ethn. Dis..

[64] Gelman A (2013). Racial disparities in human papillomavirus vaccination: Does access matter?. J. Adolesc. Health.

[65] Tabatabai MA (2014). Disparities in cervical cancer mortality rates as determined by the longitudinal hyperbolastic mixed-effects type II model. PLoS ONE.

[66] White MC, Shoemaker ML, Benard VB (2017). Cervical cancer screening and incidence by age: Unmet needs near and after the stopping age for screening. Am. J. Prev. Med..

[67] White MC (2014). Age and cancer risk: A potentially modifiable relationship. Am. J. Prev. Med..

[68] Romano AD (2010). Oxidative stress and aging. J. Nephrol..

[69] Liguori I (2018). Oxidative stress, aging, and diseases. Clin. Interv. Aging.

[70] Klaes R (1999). Detection of high-risk cervical intraepithelial neoplasia and cervical cancer by amplification of transcripts derived from integrated papillomavirus oncogenes. Cancer Res..

[71] Cullen AP (1991). Analysis of the physical state of different human papillomavirus DNAs in intraepithelial and invasive cervical neoplasm. J. Virol..

[72] Hudelist G (2004). Physical state and expression of HPV DNA in benign and dysplastic cervical tissue: Different levels of viral integration are correlated with lesion grade. Gynecol. Oncol..

[73] Pett M, Coleman N (2007). Integration of high-risk human papillomavirus: a key event in cervical carcinogenesis?. J. Pathol..

[74] Chen Wongworawat Y (2016). Chronic oxidative stress increases the integration frequency of foreign DNA and human papillomavirus 16 in human keratinocytes. Am. J. Cancer Res..

[75] Cougot D, Neuveut C, Buendia MA (2005). HBV induced carcinogenesis. J. Clin. Virol..

[76] Hu X (2010). DNA double-strand breaks, potential targets for HBV integration. J. Huazhong Univ. Sci. Technolog Med. Sci..

[77] Petersen J (1997). Increase in the frequency of hepadnavirus DNA integrations by oxidative DNA damage and inhibition of DNA repair. J. Virol..

[78] Williams VM (2014). Human papillomavirus type 16 e6* induces oxidative stress and DNA damage. J. Virol..

[79] Wei L (2009). Nitric oxide induces early viral transcription coincident with increased DNA damage and mutation rates in human papillomavirus-infected cells. Cancer Res..

[80] Wei L (2014). Tobacco exposure results in increased E6 and E7 oncogene expression, DNA damage and mutation rates in cells maintaining episomal human papillomavirus 16 genomes. Carcinogenesis.

[81] Valko M (2006). Free radicals, metals and antioxidants in oxidative stress-induced cancer. Chem. Biol. Interact..

