# Supplementary material for: Oxidative stress markers in patient-derived non-cancerous cervical tissues and cells
Source: Sci Rep. 2020 Nov 4;10:19044. doi: 10.1038/s41598-020-76159-2 (PMC7642372; doi:10.1038/s41598-020-76159-2)
Supplement: Supplementary file 1 — Supplementary Information1 [file 41598_2020_76159_MOESM1_ESM.docx]

**Supplementary Figures**

**
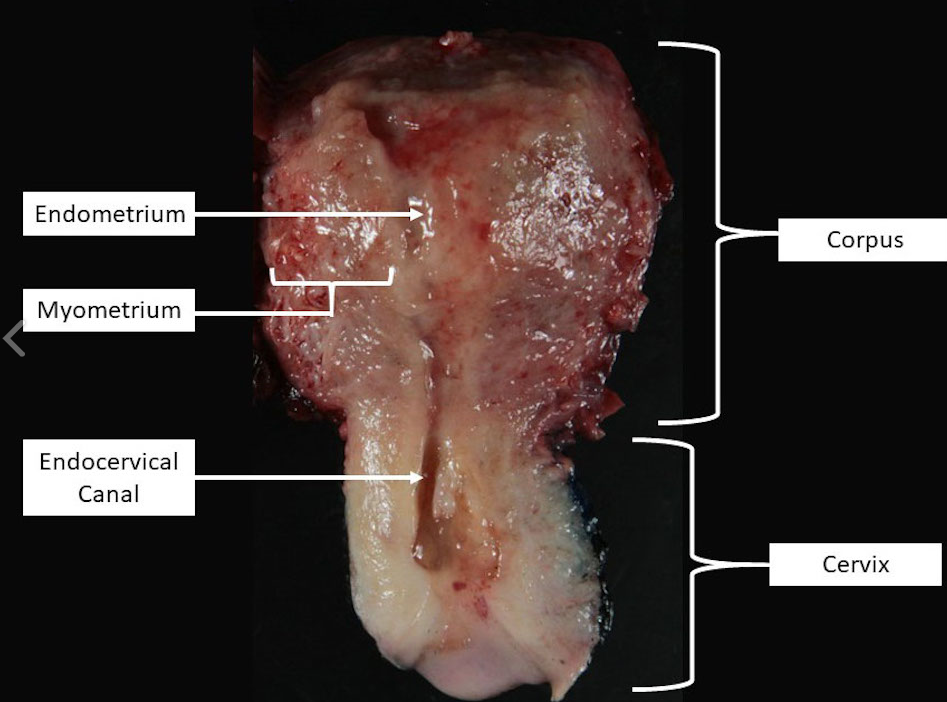
**

Transformation zone

Ectocervix

**Supplementary Figure 1 –** The gross anatomy of the human uterus. The figure, adapted from Kyle Devins, MD (<https://www.pathologyoutlines.com/topic/uterusnormal.html>), is modified to include the transformation zone and the ectocervix at the cervix.

**
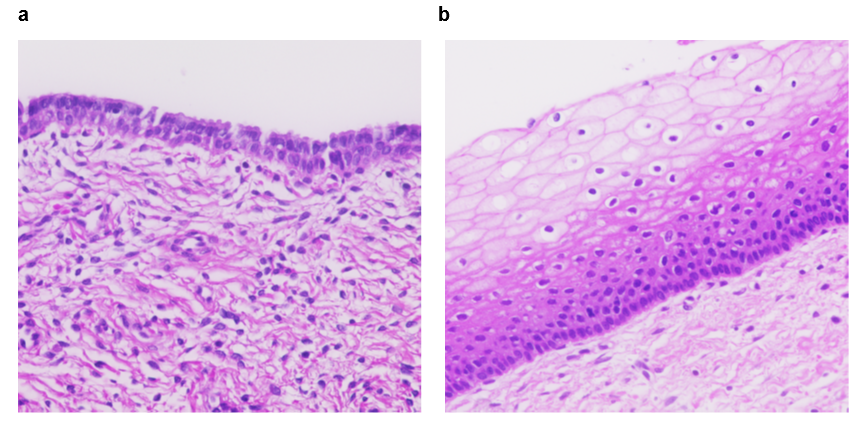
**

**Supplementary Figure 2 -** Representative TZ (a) and EC (b) tissues isolated from one of the cervical specimens. After dissection, the tissues were fixed in 4% paraformaldehyde, embedded in paraffin, sectioned and stained with hematoxylin-eosin.

**
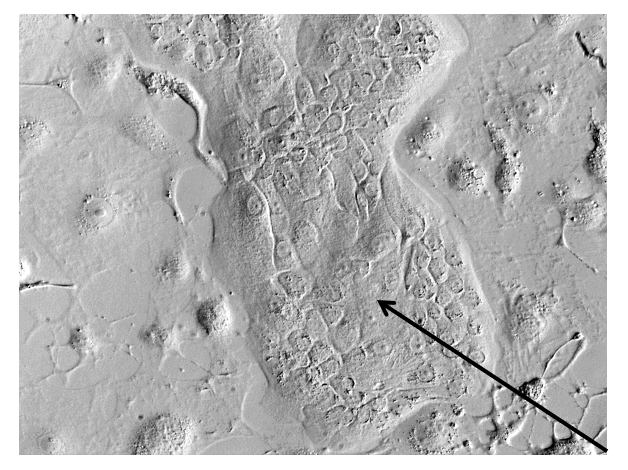
**

**Supplementary Figure 3 -** Morphology of cultured keratinocytes isolated from a TZ tissue. The arrow indicates a cluster of keratinocytes. NIH 3T3 feeder cells can be seen around the primary keratinocytes.

**S**
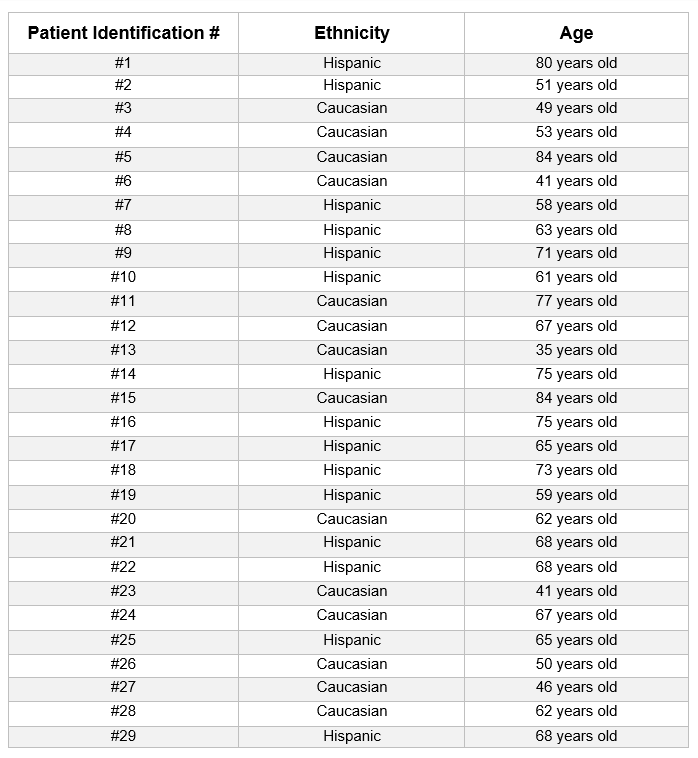
**upplementary Table 1 -** Patient identification numbers with their corresponding ethnicity and age at which they had undergone vaginal hysterectomy.
